# A Room-Temperature Operation Formaldehyde Sensing Material Printed Using Blends of Reduced Graphene Oxide and Poly(methyl methacrylate)

**DOI:** 10.3390/s151128842

**Published:** 2015-11-13

**Authors:** Wen-Yu Chuang, Sung-Yuan Yang, Wen-Jong Wu, Chih-Ting Lin

**Affiliations:** 1 Graduate Institute of Electronics Engineering, National Taiwan University, Taipei 10617, Taiwan; E-Mail: d99943047@ntu.edu.tw; 2 Department of Engineering Science and Ocean Engineering, National Taiwan University, Taipei 10617, Taiwan; E-Mails: onion781227@gmail.com (S.-Y.Y.); wjwu@ntu.edu.tw (W.-J.W.)

**Keywords:** RGO, PMMA, formaldehyde, gas sensor

## Abstract

This work demonstrates a printable blending material, *i.e.*, reduced graphene oxide (RGO) mixed with poly(methyl methacrylate) (PMMA), for formaldehyde sensing. Based on experimental results, 2% RGO/10% PMMA is an optimal ratio for formaldehyde detection, which produced a 30.5% resistance variation in response to 1000 ppm formaldehyde and high selectivity compared to different volatile organic compounds (VOCs), humidity, CO, and NO. The demonstrated detection limit is 100 ppm with 1.51% resistance variation. Characterization of the developed formaldehyde sensing material was performed by Fourier-transform infrared (FTIR) spectrometry, scanning electron microscopy (SEM), and Raman spectroscopy. Based on Raman spectroscopy, the basic sensing mechanism is the band distortion of RGO due to blending with PMMA and the adsorption of formaldehyde. This work establishes insights into the formaldehyde sensing mechanism and explores a potential printable sensing material for diverse applications.

## 1. Introduction

Formaldehyde is one of the most widespread volatile organic compounds (VOCs) in our life. It is widely used in the chemical industry, household materials, paints, wooden materials, adhesives, cosmetics, and textile industry [1,2,3,4,5]. According to the U.S. Environmental Protection Agency (EPA) and International Agency for Research on Cancer (IARC), it is categorized as cancerogenic [6,7,8]. Exposure to formaldehyde leads to different diseases [9,10,11]. It also causes damage to the lymphatic and central nervous systems [12]. In the Occupational Safety and Health Administration (OSHA) regulations, the exposure limit for formaldehyde in the workplace is 0.75 ppm during 8 h, and 2 ppm is the maximum exposure during a 15-min period.

Different methods such as chromatography [13], spectrophotometry [14], differential optical absorption spectroscopy (DOAS), Fourier transform infrared absorption, and laser induced fluorescence spectroscopy (LIFS), have been developed for monitoring formaldehyde. For small-size and real-time monitoring devices, in addition, many microscale gaseous formaldehyde detection systems [15] and various metal oxide semiconductor materials, such as SnO_2_ [16,17,18], doped ZnO [19,20,21], CdO/In_2_O_3_ [22], and nanostructured materials [23] were also investigated. These sensing technologies offer good sensitivity to formaldehyde, however, most of them require bulky and expensive equipment. In addition, most of them also have high-power consumption due to their high temperature operation. To address the need for small-size and low-power characteristics for gas sensing applications, room-temperature operation and low-cost printable sensing materials have the potential to be integrated in multi-functional sensor systems [24].

Graphene is widely used for gas sensing because of its 2D structure and high electronic mobility which offer characteristic low noise and low detection limits [25,26]. To enhance the gas adsorption and sensing capabilities, previous works have developed graphene-based sensing materials by functionalization with polymers, metals, or other modifiers to produce dangling bonds on its surface [27,28]. Since surface functionalization determines the exfoliation behavior of graphene oxide (GO) [26], different gas sensors based on reduced graphene oxide (RGO) materials have been investigated, such as NO_2_ [29], CO_2_ [30], and organic vapor sensing devices [31,32,33]. For formaldehyde detection, the blending of RGO/PMMA was proposed because of the high solubility of formaldehyde in PMMA [32]. However, the sensing mechanism and characteristics of RGO/PMMA blends in sensing formaldehyde are not well understood. In this work, therefore, the sensing properties of printable RGO/PMMA blends for formaldehyde monitoring are further characterized by scanning electron microscopy (SEM), FTIR, and Raman spectroscopy and discussed.

## 2. Experimental Section

### 2.1. Materials 

RGO (product number N002-PDR) was purchased from Angstron Materials (Dayton, OH, USA). PMMA (m.w. 15,000) and formaldehyde solution (formaldehyde, 37 wt% solution in water with 5%–15% methanol) were obtained from ACROS (Thermo Fisher Scientific, Waltham, MA, USA). Tetrahydrofuran (THF, 99%) was purchased from ALD (Taipei, Taiwan).

### 2.2. Device Fabrication

For microelectrode fabrications, a p-type silicon wafer with a 300 nm thickness of silicon oxide on the surface was used as the device substrate. After various cleaning processes, *i.e.*, washing with acetone, isopropyl alcohol (IPA), and DI water, the substrate was dried under nitrogen gas and heated at 120 °C for 10 min to remove any residual humidity on the surface. Utilizing photolithography, the width/length of the sensing electrode was defined at a ratio of 800/40 µm. Fabricated Cr/Au electrodes with thickness of 20 nm/200 nm were produced by e-gun evaporation and lift-off processes.

### 2.3. Analysis

To characterize the developed RGO/PMMA sensing material, FTIR, SEM, and Raman spectroscopy were used to analyze the sensing mechanism of RGO/PMMA blends and the interaction between RGO/PMMA and formaldehyde. A FTIR instrument (MB-series, BOMEM, QC, Canada) with a resolution of 0.5 cm^−1^ and a frequency range 350–7800 cm^−1^ was used for material analysis. SEM (JSM-6700F, JEOL, Tokyo, Japan) with a resolution of 1.0 nm (15 KV) at a probe current from 10^−13^ to 2 × 10^−9^ A was used for morphology analysis. A micro-Raman setup consisting of a Raman instrument (Almega XR from Nicolet, Boston, MA, USA) and a microscope (BX51 from Olympus, Center Valley, PA, USA) with a laser wavelength of 780 nm and total energy of 100 mW was used for material analysis.

### 2.4. PMMA/RGO Sensing Film Preparation

To obtain PMMA/RGO sensing film composition ratios, different w/w ratios of RGO, such as 1%, 2% and 3%, were dispersed in 1 g of THF. RGO solutions were placed in an ultrasonicator for 3 h to improve the dispersion characteristics. Separately, different w/w ratios of PMMA, such as 1%, 2%, 5%, 10%, 20%, and 30%, were also dissolved in 1 g of THF and put in an ultrasonicator for 3 h. The PMMA/RGO sensing film was then preparing by adding RGO solution slowly into PMMA solution. After the preparation of PMMA/RGO blending solutions, the sensing films were obtained by spin coating with the optimal condition of 1500 rpm for 30 s and then dried at room temperature for 1 h.

### 2.5. Characteristics and Measurements

To measure the response of the developed sensing material, a LCR meter (E4980A, Agilent, Taipei, Taiwan) was used to provide 1 V of DC bias voltage and measure the resistance of the sensing film. Utilizing the interface program of a computer, the resistance readout of the sensing films can be obtained. For gas chamber controls, on the other hand, a vacuumed pumping step followed by N_2_/air flushing was used to remove ambient gases in the testing gas chamber. Then the chamber was evacuated again to 5 × 10^−2^ torr. This was set as the initial conditions.

In formaldehyde detection experiments, the purchased 37 wt% formaldehyde solution was diluted with DI water to specific w/w concentrations. Then the designated concentration of formaldehyde solution was injected with a background gas, such as N_2_ or dry air, into the testing chamber and continuously measured for 15 min. Because the testing chamber was under vacuum, the solution was vaporized within the chamber. To obtain accurate concentrations of the injected formaldehyde, it was measured with Gastec Formaldehyde Detector Tubes from Zefon (Ocala, FL, USA). Between each formaldehyde concentration measurement, the chamber was cleaned as previously described to restore the initial conditions. In selectivity measurements, following the same experimental procedure described above, humidity, CO_2_, NO, CO and different kinds of VOC gases were tested. For NO, CO, and CO_2_ detection, gas cylinders of specific concentrations were used. For humidity and VOC, on the other hand, a similar procedure as followed for formaldehyde was used to inject different solutions into the testing chamber. In these experiments, the humidity concentration was measured by a commercial humidity sensor, which has a built-in SHT11 (Sensirion, Staefa, Switzerland) sensor chip. The VOC concentration was estimated by ideal gas behavior under the conditions of 25 °C and a chamber volume of 50 L. A completely vaporized liquid injection was assumed [33]. In addition, the pressure effect was also tested to distinguish the formaldehyde sensing response from pressure effects. 

## 3. Results and Discussion

### 3.1. SEM and FTIR Analysis

**Figure 1:**
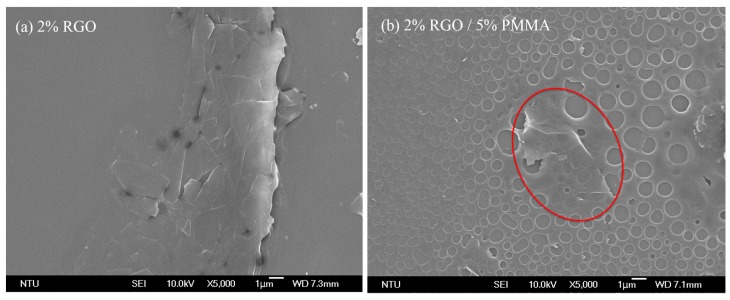

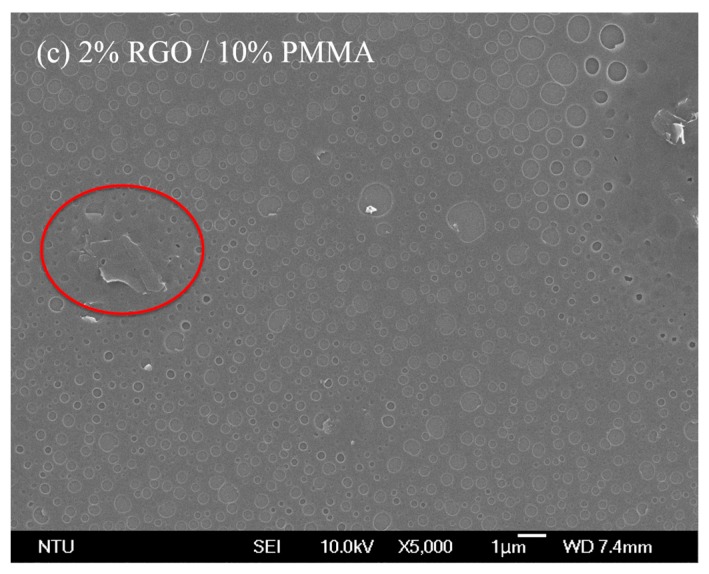
SEM pictures of different RGO/PMMA sensing film compositions: (**a**) 2% RGO, (**b**) 2% RGO/5% PMMA, and (**c**) 2% RGO/10% PMMA composite. The red circles indicate the graphene sheets.

To investigate the morphology of the developed RGO/PMMA sensing film, SEM with 1 nm resolution at 15 kV is performed as shown in Figure 1. Figure 1a represents flakes of graphene thin film. RGO/PMMA blends formed porous film surfaces as shown in Figure 1b,c. The porous structure formed during the solvent evaporation process, which is a competition between the phase separation dynamics and the solvent evaporation rate [33]. The membrane becomes porous as the solvent-rich phase is dried out. Comparing Figure 1b, *i.e.*, 2% RGO with 5% PMMA, and Figure 1c, *i.e.*, 2% RGO with 10% PMMA, it is clear that RGO is gradually covered by PMMA as the composition ratio of PMMA increases. Since the sensing mechanism occurs at the interface of RGO and PMMA, as previously mentioned, the higher composition ratio of PMMA in the RGO/PMMA sensing film leads to less sensing response to formaldehyde.

**Figure 2:**
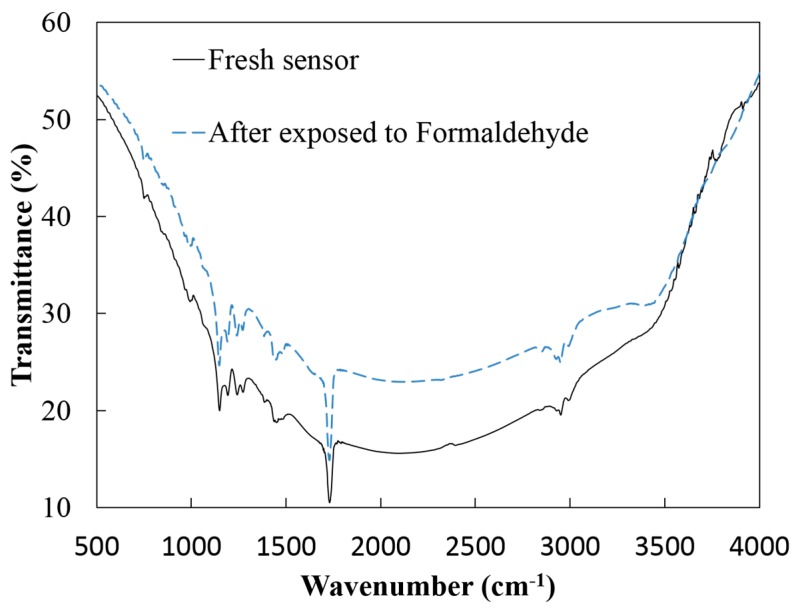
FTIR analysis for fresh RGO/PMMA based sensor (black line) and after sensor exposed to formaldehyde (dashed line).

Figure 2 presents FTIR analysis of 2% RGO/10% PMMA sensing film before and after the exposure to formaldehyde. Before the sensor was exposed to formaldehyde only a peak at 3496 cm^−1^ for the -OH groups representing the incomplete RGO is observed [31]. Peaks at 2960 and 3000 cm^−1^ for C-H stretching, 1742 cm^−1^ for C=O stretching, and 1203 cm^−1^ for C-O-C stretching can be observed in PMMA [34]. After the sensor is exposed to formaldehyde, the spectrum is similar as the one before exposure to formaldehyde. In other words, there are no additional peaks after the exposure toformaldehyde. Based on this result, it can be confirmed that there are no chemical reactions when RGO/PMMA is exposed to formaldehyde, which is evidence of physical adsorption between RGO/PMMA and formaldehyde.

### 3.2. Sensor Characterization for Formaldehyde Detection

**Figure 3:**
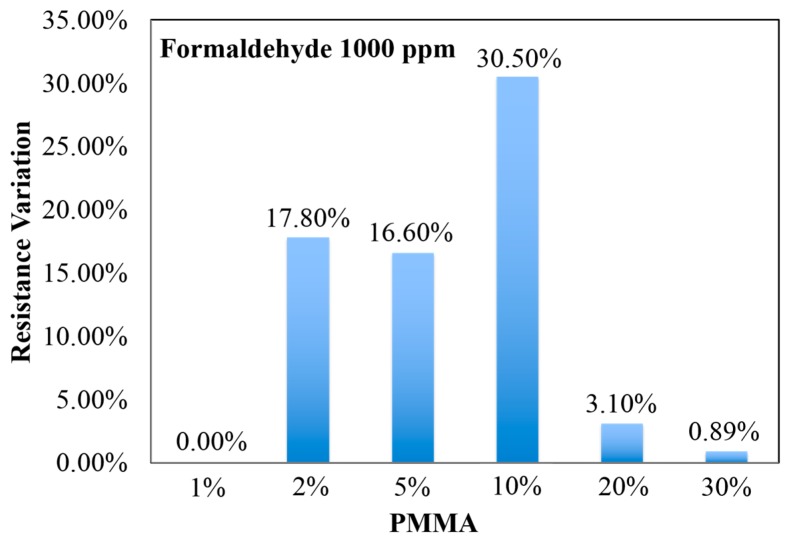
The formaldehyde sensing result of different PMMA blending concentrations with 2% RGO.

The sensor response was defined as (R_g_ − R_0_)/R_0_ × 100%. Based on the defined sensor characteristic, the sensing response of RGO/PMMA sensing material has only 0.30% resistance variation between N_2_ or an air environment (1 atm) and a vacuum environment (10^−2^ torr). As a consequence, the formaldehyde-detection baseline of RGO/PMMA sensing material can be established. Since formaldehyde is in a solution form with water and methanol, the maximum testing solution volume, *i.e.*, 0.4 mL, of water and methanol are tested to obtain the sensor responses to these background liquids. The sensor response to the background liquid are 0.3% (for methanol) and 0.56% (for water). These responses are similar to the noise level (0.3%). The blend of RGO and PMMA improved the selectivity because of the good solubility of formaldehyde in PMMA [31]. Although PMMA enhances formaldehyde adsorption, it also covers the RGO and masks effective conducting paths. This leads to less sensitivity to formaldehyde, *i.e.*, less resistance variance. Furthermore, RGO is a conductive material, so the resistance of pure 2% RGO, which is about 50 Ω, is very low. On the other hand, the resistance of 10% PMMA is higher than the gigaohm that our LCR meter can detect. Therefore, the resistance is highly dependent on the ratio of RGO and PMMA, *i.e.*, the resistance of 2% RGO/10% PMMA is about 240 ± 15 Ω. To obtain an optimal ratio of RGO/PMMA for formaldehyde detection, as shown in Figure 3, different blending ratios of RGO/PMMA were examined under 1000 ppm formaldehyde. Based on this experimental result, 2% RGO blended with 10% PMMA produces a 30.50% resistance variation in response to 1000 ppm formaldehyde, which is larger than other ratios. 

**Figure 4:**
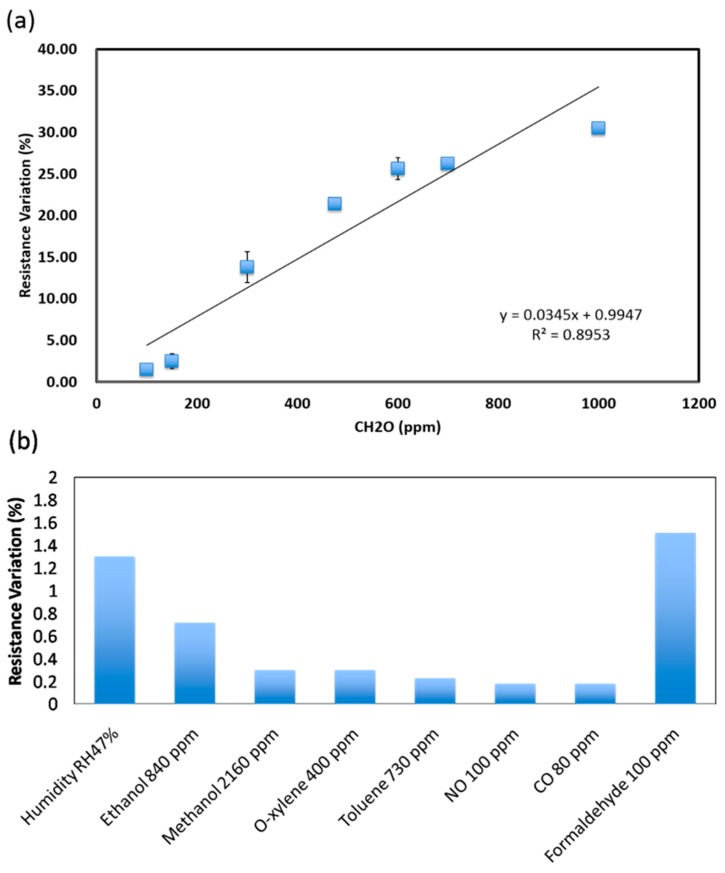
The characteristics of 2% RGO/ 10% PMMA sensing material operated at room temperature for: (**a**) formaldehyde sensitivity and (**b**) selectivity.

Utilizing this optimal blending ratio, the formaldehyde sensitivity curve can be determined as shown in Figure 4a. The lowest detectable formaldehyde concentration is 10 ppm (0.50% resistance variation), a response similar to the resistance variation caused by pressure changes and background liquid. Therefore, the limitation of formaldehyde sensing is estimated at 100 ppm with about 150 s and 180 s of response and recovery time, respectively. The higher concentration leads to a shorter response time and longer recovery time.

Furthermore, the selectivity test results of the developed printable RGO/PMMA sensor can be seen in Figure 4b. Compared with the sensor response to 100 ppm formaldehyde, it is obvious that the developed RGO/PMMA has less response to common VOC gases, such as ethanol, *o*-xylene, toluene, and methanol. Although the response to 840 ppm ethanol is higher than the noise level, the ethanol response is still two times smaller than the 100 ppm formaldehyde response. Furthermore, the sensor responses to 100 ppm NO and 80 ppm CO are also around noise level. Therefore, the developed sensing material shows a relatively good selectivity for formaldehyde. The performance of previously developed formaldehyde gas sensors is summarized in Table 1. Compared to metal oxide-based materials, the room temperature operated polymer-based sensing materials offer low-power consumption and are suitable for integration with wireless networks for IoT applications. Although it can be noted that the limit of detection of the developed RGO/PMMA sensor is enough for formaldehyde detection in workspace monitoring, based on our understanding of the sensing mechanism, it could be improved for future applications. Compared with polymer-based sensing materials [35], on the other hand, the developed RGO/PMMA has low-cost solution manufacturing advantages.

**Table 1:** Performance of some formaldehyde gas sensors.

Year [Reference]	Sensing Material(s)	Working Temperature	Sensitivity	Sensing Range
2011 [22]	ZnO	400 °C	0.564 ppm^−1^ (R_a_/R_g_)	1–1000 ppm
2011 [24]	Cd activated Sn-ZnO	200 °C	10 ppm^−1^ (R_a_/R_g_)	1–205 ppm
2014 [35]	Polyaniline	25 °C	0.21% ppm^−1^ (△R/R_0_)	0.4–400 ppm
2015 [23]	In_2_O_3_/ZnO	300 °C	0.3 ppm^−1^ (R_a_/R_g_)	100–2000 ppm
2015 [19]	Pd-SnO_2_	160 °C	0.188 ppm^−1^ (R_a_/R_g_)	5–1000 ppm
This Work	RGO/PMMA	25 °C	0.043% ppm^−1^ (△R/R_0_) 0.007 ppm^−1^ (R_g_/R_0_)	10–1000 ppm

### 3.3. Raman Spectroscopy

**Figure 5:**
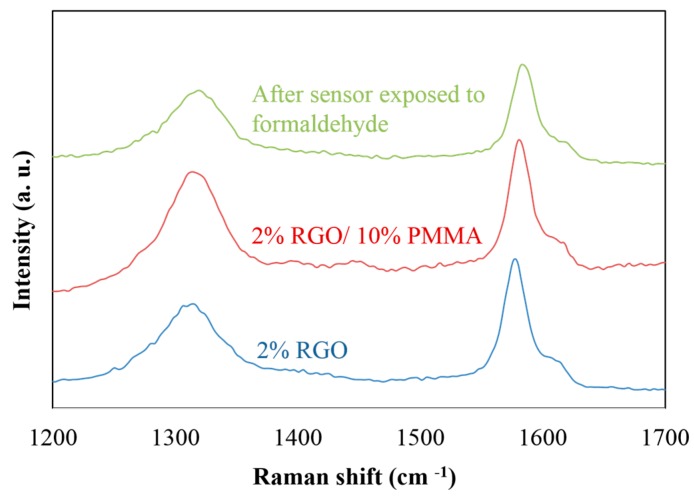
Raman spectroscopy (E_L_ = 1.59 eV and λ_L_ = 780 nm) of the RGO-based sensing materials.

To further explore the effect of formaldehyde adsorption in RGO/PMMA thin film, Raman spectroscopy was used. In Figure 5, the D peak and G peak are due to sp^2^ bonded carbon materials. The D peak results from the breathing modes of rings. It shows the disorder of the structure, *i.e.*, the more disordered the structure, the higher the intensity of the D peak [36]. On the other hand, the G peak is due to the bond stretching of all pairs of sp^2^ atoms in both rings and chains. The intensity of the G peak depends on the layers of carbon material, *i.e.*, more layers of carbon material lead to higher intensity of the G peak. Based on these statements, Figure 5 shows that the blending of PMMA and the adsorption of formaldehyde led to the increase in D band intensity, which means the more disordered RGO electron structures. Moreover, the ratio of D and G intensities can be used to calculate the graphite crystal domain size, *L_a_*, which can be determined from the following general formula:

(1) $$L_{a}{\;}^{-1}\left({\text{nm}}^{-1}\right)=\;\frac{{\text{E}}_{\text{L}}^{4}}{560}(\frac{I_{D}}{I_{G}})$$

In other words, *L_a_* increased inversely with the ratio of the D and G intensities [37].

**Figure 6:**
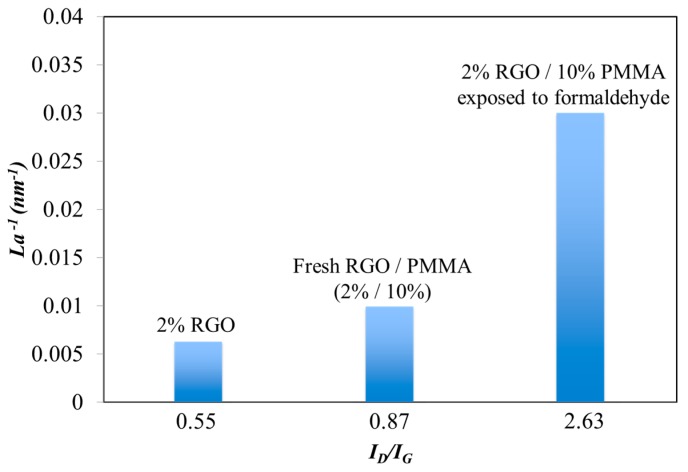
Dependency of the *I_D_/I_G_* ratio and the inverse values of crystallite diameter for RGO-based sensing films.

In the Raman experiment, the excitation energy, E_L_ is 1.59 eV (λ = 780 nm). Figure 6 shows the plot of the intensity ratio of D and G bands (*I_D_/I_G_*) *versus 1/L_a_* for various RGO-based sensing films. The blending of PMMA leads to a *I_D_/I_G_* ratio increase from 0.55 to 0.87. This confirms that the resistance difference between RGO/PMMA and pure RGO can be a result of the disordering of the RGO electron structure. Based on this observation, the RGO structure disorder results in the resistance increase of RGO/PMMA. Furthermore, the *I_D_/I_G_* increased to 2.63 after the adsorption of formaldehyde. The raise of *I_D_/I_G_* could be explained by the adsorption of formaldehyde enlarging the distortion of the RGO electron structures, resulting in the increase of resistance. 

## 4. Conclusions

In this work, the characteristics of a printable RGO/PMMA sensing material for formaldehyde detection have been investigated. Based on the experimental results, 2% RGO blended with 10% PMMA is the optimal ratio for formaldehyde sensing, which produced a 30.50% resistance variation for 1000 ppm formaldehyde and a lower detectable concentration of 100 ppm with 1.51% resistance variation. Furthermore, this sensor displayed good selectivity for formaldehyde compared to other VOCs, CO, and NO. In addition, the physical and chemical characteristics of the sensing film were also explored based FTIR, SEM, and Raman analysis investigations. These analyses show that the blending of PMMA not only improved the adsorption of formaldehyde but also increased the distortion of the RGO electron structure. Moreover, the adsorption of formaldehyde further increased the distortion of the electron structure in RGO. However, the adsorption of formaldehyde also breaks down the physical interaction between PMMA and RGO, and as a consequence, it degrades the formaldehyde sensing sensitivity.
